# The impact of acute stress on hormones and cytokines, and how their recovery is affected by music-evoked positive mood

**DOI:** 10.1038/srep23008

**Published:** 2016-03-29

**Authors:** Stefan Koelsch, Albrecht Boehlig, Maximilian Hohenadel, Ines Nitsche, Katrin Bauer, Ulrich Sack

**Affiliations:** 1 Max Planck Institute for Human Cognitive and Brain Science, Stephanstr. 1a, 04103 Leipzig, Germany; 2 Department of Biological and Medical Psychology, University in Bergen, Jonas Liesvei 91, 5009 Bergen, Norway; 3 Institute of Clinical Immunology, Medical Faculty, University of Leipzig, Johannisallee 30, 04103 Leipzig, Germany; 4 Clinic for gastroenterology and rheumatology, Medical Faculty, University of Leipzig, Liebigstr. 20, 04103 Leipzig, Germany

## Abstract

Stress and recovery from stress significantly affect interactions between the central nervous system, endocrine pathways, and the immune system. However, the influence of acute stress on circulating immune-endocrine mediators in humans is not well known. Using a double-blind, randomized study design, we administered a CO_2_ stress test to *n* = 143 participants to identify the effects of acute stress, and recovery from stress, on serum levels of several mediators with immune function (IL-6, TNF-α, leptin, and somatostatin), as well as on noradrenaline, and two hypothalamic–pituitary–adrenal axis hormones (ACTH and cortisol). Moreover, during a 1 h-recovery period, we repeatedly measured these serum parameters, and administered an auditory mood-induction protocol with positive music and a neutral control stimulus. The acute stress elicited increases in noradrenaline, ACTH, cortisol, IL-6, and leptin levels. Noradrenaline and ACTH exhibited the fastest and strongest stress responses, followed by cortisol, IL-6 and leptin. The music intervention was associated with more positive mood, and stronger cortisol responses to the acute stressor in the music group. Our data show that acute (CO_2_) stress affects endocrine, immune and metabolic functions in humans, and they show that mood plays a causal role in the modulation of responses to acute stress.

The interactions between the central nervous system, the endocrine system, and the immune system are subjects of intense investigation in the field of biomedical science^1^. Particularly, effects of stress (and recovery from stress) on immune function have received a vast amount of attention due to their impact on human health and well-being. However, there is lack of information regarding the influence of acute (transient) stress on circulating immune-endocrine mediators such as hormones and cytokines in healthy individuals. Investigating such influence is challenging, due to the fact that effects of stress are difficult to differentiate from confounding factors such as exercise, current health status, psychological states (e.g., depressed mood and anxiety), and inter-individual variability in responsiveness to stress^2^,^3^. Moreover, such alterations of endocrine and immune parameters are usually moderate and mostly stay within the reference values of healthy persons, although they can be precisely detected^4^. To obtain sufficient statistical power in the face of these methodological difficulties, our study employed a CO_2_ stress test, high sensitivity laboratory tests, and a relatively large population of participants (*n* = 143) to identify the effects of acute stress on hormonal and immunological parameters^5^.

The CO_2_ stress test used in the present study (inhalation of 35% CO_2_) is a test originally employed as an experimental model of panic^6^. The CO_2_ test incites noticeable vegetative reactions (such as changes in heart rate and blood pressure)^6^, an increase of serum noradrenaline and salivary alpha-amylase^7^, as well as an increase in serum cortisol^6^. The CO_2_ test has therefore been taken to represent an acute physiological stressor suited to model the human stress response^6^. Thus, purely physiological effects of stress can be investigated, independent of physical stressors (e.g., exercise) or psychological stressors (e.g., mental stress). Whether CO_2_ stress also affects immune parameters is unknown, although knowledge about effects of physiological stress on immune function is important for the understanding of neuro-endocrino-immunological function.

To investigate this, we measured effects of CO_2_ stress on several mediators with immune function, in addition to two hypothalamic–pituitary–adrenal (HPA) axis hormones (adrenocorticotropic hormone [ACTH] and cortisol), and on serum noradrenaline (released into the blood stream by cells of the adrenal medulla in response to stimulation by sympathetic preganglionic neurons). The mediators with immune function included two cytokines (interleukin-6 [IL-6] and tumor necrosis factor [TNF]-α), leptin (which also acts as pro-inflammatory peptide)^8^, and somatostatin (which down-modulates a number of immune functions, such as lymphocyte proliferation, immunoglobulin production and the release of pro-inflammatory cytokines)^9^. Generally, it is assumed that stress-related HPA and sympathetic nervous system activity inhibits the functions of inflammatory cells, in particular cytokines (such as IL-6 and TNF-α)^10^. Whether this mechanism is also activated during acute stress has remained elusive in previous research. This lack of knowledge is surprising, given that acute stress in everyday life might have an impact on immune system activity (in both positive and negative ways)^11^, and thus on an individual’s health.

Moreover, to investigate latencies of peak responses, and to shed light on the recovery of parameters affected by the stress test, we obtained serum values of these parameters not only directly before and after the CO_2_ stress test, but also at several time points following the stress test (see also Fig. 1). This was intended to provide useful information about the most relevant time points for sample collection in future studies.

Another aim was to investigate the influence of mood as a psychological factor on endocrine and immune reactions to stress and recovery from stress. As a mood-induction procedure we used positive music in one study group, and an acoustical control stimulus in a control group. Music as a stimulus was chosen because music has been shown to be a potent mood modulator^12^,^13^, and because music-evoked engagement of neurochemical systems underlying stress and immunity has received increasing interest during the past years (see, e.g, refs 14, 15, 16, 17 and below).

Several laboratory studies with healthy participants reported cortisol reductions in response to music (without a stressor), compared to a control group^18^,^19^,^20^,^21^. In general, it seems that a decrease in cortisol is more likely during tranquilizing music, a reduction in negative mood, and in music therapy settings, whereas an increase in cortisol levels is more likely when music is perceived as vitalizing, or when individuals are physically active^14^,^22^. In addition, several studies measured salivary cortisol levels following a stressor^23^,^24^,^25^. Two studies observed *lower* cortisol levels in a music listening group (compared to silence group)^24^,^26^, and one study reported *higher* cortisol levels post-stressor in a music group (compared to a group listening to the sound of rippling water)^25^. Thus, the effects of mood (evoked by music) on cortisol responses to stress are not well understood, at least not in the context of laboratory studies. In clinical settings, listening to music before, during, and after medical interventions has been reported to correlate with lower cortisol levels, associated with reductions of anxiety (for reviews see refs 15,27).

With regard to immune-parameters, there is scarcity of music studies investigating effects of music on cytokines (for reviews see refs 28,15,16), and to our knowledge only two studies with a control-group design investigated effects of music on Immunoglobulin A^29^,^14^. Our study aims at shedding more light on effects of music on immune functions, and interactions between cortisol and immune parameters. Insights into psychological mechanisms associated with immune function are particularly relevant with regard to mood disorders, such as depression, and with regard to somatic diseases with affective components, such as chronic diseases of the immune system^30^,^31^,^32^.

We addressed the following four core hypotheses in our study: (1) Acute CO_2_-induced stress leads to an increase of serum ACTH, cortisol, and noradrenaline; (2) even short-term (acute) stress induces inflammatory activity involving IL-6^33^, TNF-α^34^, and somatostatin^35^; (3) beyond these HPA, sympathetic, and inflammatory parameters, we expected an increase of serum leptin in response to acute stress (due to its role as mediator of stress-induced obesity)^36^; (4) finally, we tested whether music-evoked positive mood has effects on both stress and immune parameters during recovery from acute CO_2_ stress.

## Methods

### Summary of study design and procedures

**Figure 1:**
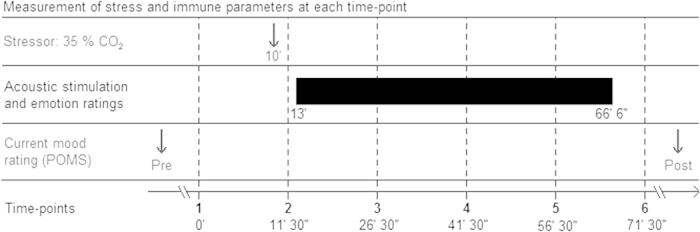
Illustration of the experimental design. In the beginning, participants (*n* = 143) rated their current mood using the Profile of Mood States (POMS, see third row). Then, the first blood sample was drawn (time point 1, bottom row), and about 10 min later the CO_2_ stress-test was administered (top row). Following the stress test, another blood sample was drawn (time point 2), and the acoustic stimulus (positive music or neutral control stimulus) was delivered (second row). Then, every 15 min, four further blood samples were obtained. After the last blood sample was obtained (at time point 6), participants rated again their mood (see third row).

In our double-blind, randomized study, acute stress was induced in *n* = 143 participants by CO_2_ insufflations. Serum levels of cortisol, ACTH, interleukin-6 (IL-6), noradrenalin (NA), leptin, somatostatin (SIH), and TNF-α were measured at six time points: directly before and after the CO_2_ challenge, and then every 15 minutes over the course of one hour (see Fig. 1). After the CO_2_ stressor, the mood of participants was systematically influenced by presenting them either with a positive music stimulus (music group) or with a neutral auditory control stimulus (control group).

### Participants

143 healthy volunteers participated in the study, all participants were students from the University of Leipzig (for descriptive statistics of the study population see Table 1). Using a computer algorithm, participants were randomly assigned either to the music group (*n* = 71, mean age = 24.8 years, range 20–33 years, 35 females and 36 males, mean body mass index 22.4) or to the control group (*n* = 72, mean age = 24.9 years, range 20–32 years, 36 females and 36 males, mean body mass index 22.2). Moreover, the computer algorithm assigned participants randomly to one of three measurement times (11:00 a.m., 2:00 p.m. and 5:00 p.m.). The computer algorithm ensured that all study groups were balanced with regard to group size, male/female ratio, mean age, mean height, mean weight, and body mass index. By balancing the study groups across three different times of day (instead of measuring at only one fixed time), we can guarantee that the effects observed not only hold for one particular time of day (e.g., morning or afternoon), but can be generalized to most time points during the day. Moreover, by balancing subjects carefully across the different times of day, we can exclude that our data are biased by the circadian rhythmicity of the measured parameters (and although this was not the focus of this study, we will report data informing about the circadian rhythmicity of the measured parameters in the Supplementary Information). Note that, as the results will show, the circadian rhythm of all of the investigated parameters did not interact with the stress test or with the recovery from stress.

Exclusion criteria were pregnancy, uncontrolled hypertension (blood pressure above 160 over 90 mmHg), chronic lung diseases (including asthma), history of psychiatric or neurologic disorders (including panic disorder, migraine or seizure disorder), or immunological disorders. None of the subjects took any medication (including anti-inflammatories or steroids), and none of the subjects had needle phobia. Written informed consent was obtained from all participants. The study was approved by the ethics committee of the University of Leipzig (#166/2006), and conducted according to the Declaration of Helsinki.

### Stress induction

Subjects took a single full vital capacity breath of a gas mixture of 35% CO_2_ and 65% O_2_. Breaths were controlled and recorded using a spirometer (ZAN 100 Flowhandy, ZAN Messgeräte GmbH, Oberthulba, Germany). The single full vital capacity breath was practised beforehand, and observed by both the participant and the experimenter on the spirometer screen.

### Procedure

Participants were not informed as to whether they were in the music or the control group. After giving written informed consent, participants filled out questionnaires on personal data, and the short (35-item) German version of the Profile Of Mood States (POMS)^37^ which consists of the four scales “Depression/Anxiety”, “Fatigue”, “Vigor” and “Irritability”. Then, blood pressure was measured, and an intravenous cannula was inserted in an antecubital vein of the left arm. Immediately after the insertion of the cannula (within the first seconds after insertion), the first blood sample was drawn (time point 1). About 10 min later, the CO_2_ stress test was administered (see also Fig. 1). Directly after the CO_2_ challenge, participants lay down in supine position, closed their eyes, and were presented via headphones either with the musical stimulus or with the control stimulus (stimuli are described below), and about 90 seconds after the CO_2_ challenge, a second blood sample was obtained (time point 2). Subsequently (while participants were listening to either the music or the control stimulus), four further blood samples were taken over the course of one hour. Thus, blood samples were drawn at six different time points (TP): Before the CO_2_ challenge (TP 1), 90 seconds after the CO_2_ challenge (TP 2), and then every 15 minutes (TP 3–6, see also Fig. 1). Note that the parameters measured at TP 1 might have been influenced by some participants’ anxiety associated with the insertion of the cannula. Thus, the serum values of TP 1 do not represent a normal baseline value. Moreover, the effects of acute stress on the parameters measured at TP 2 (taken directly after the CO_2_ stress test) might have been due to both the CO_2_ stress test and in part the venipuncture^38^. These issues will be discussed further in more detail below.

Experimenters were blinded with regard to which of the two stimuli the participant was exposed to. Likewise, participants were only informed that an auditory stimulus would be presented to them, and they were asked to tap the beat of the stimulus with their right index finger, allowing us to guarantee that subjects of both groups paid similar attention to the stimulus, and engaged in a similar way with the task (a procedure that has been employed successfully in previous studies, e.g. refs 39,40). After each auditory stimulus, there was a silent interval of 40 seconds in which participants opened their eyes and provided ratings on how they felt during the last piece regarding valence and emotional arousal. Ratings were obtained by using 9-point scales. As the results will show, the control stimulus was perceived as neither pleasant nor unpleasant. Thus, our control condition might also provide a valuable method for the investigation of recovery from stress in general, perhaps being even more adequate than simply letting participants relax in silence (see also Discussion). Directly after the very last auditory stimulus, participants filled out the POMS mood-questionnaire again (to assess effects of the experiment on the mood of participants). Finally, participants were debriefed.

### Biochemical analyses

Blood samples were cooled and centrifuged immediately after the respective sample was taken. Plasma and serum samples were stored at −80° C until analysis. The following parameters were analyzed using test kits according to the manufacturers’ guidelines (manufacturers and detection limits are listed in parentheses): cortisol (IBL, Hamburg, Germany; 2.4 ng/ml); ACTH (Biomerica, Newport beach, CA; 0.46 pg/ml); Interleukin-6 (IL-6; R&D Systems, Minneapolis, MN; 0.11 pg/ml); Noradrenaline (NA; Labor Diagnostika Nord, Nordhorn, Germany; 0.2 ng/ml), Leptin (R&D Systems, Minneapolis, MN; 7.8 pg/ml); Somatostatin (SIH; Peninsula Laboratories LLC, San Carlos; 10 pg/ml); and Tumor Necrosis Factor (TNF)-α (R&D Systems, Minneapolis, MN; 0.1 pg/ml). Extreme values (i.e., values above or below 2 SD of the mean) of plasma and serum parameters were regarded as artefacts and excluded from the statistical analysis (~4% of all data).

### Music and control stimuli

Stimuli were taken from a previous study^12^. For the music group, 18 pieces of instrumental music (without lyrics) from various styles and epochs were used as stimuli (Classical, Jazz, Irish folk, South American, Reggae). Range of beats per minute was 106–132. These pieces had been shown to evoke feelings of pleasure and happiness^12^. The control group was presented with 18 computer-generated pieces that were melodic sequences of random tones of the chromatic scale, presented in isochronous intervals. All twelve pitch classes of the chromatic scale occurred with equal probability in these pieces (that is, pieces were not tonal, and had no tonal centre). The control stimuli had the same pitch range, mean pitch, tempo and duration as the musical comparison stimuli. Moreover, the timbres of the control stimuli were similar to those of the respective musical stimulus (e.g., for a musical piece with flutes, the corresponding control stimulus was created with a flutes-timbre). Control stimuli were synthesized with Reason 3.0 (Propellerhead Software, Stockholm, Sweden). In the previous study that also used these stimuli (ref. 12), participants rated the felt emotional valence of these stimuli as neutral, that is, they neither liked nor disliked the control stimulus. Moreover, in that study ratings of felt physiological arousal evoked by music and control stimuli did not differ^12^. Duration of each set of stimuli (music and control stimuli) was ~41 minutes.

### Data Analysis

Effects of stress test, group (music/control), time of day, and sex, as well as interactions between variables were calculated using MANOVAs with *time-point* (six time points, see also Fig. 1) as within-subjects factor, and *stimulus group* (music, control), *sex* (male, female), and *time of day* (11:00 a.m., 2:00 p.m., 5:00 p.m.) as between-subjects factors. Bonferroni-corrected significance threshold for main effects was *p* = 0.005. Statistical analysis was performed using PASW Statistics 18 (IBM Deutschland GmbH, Ehningen, Germany). Parametric tests were computed because none of the parameters deviated significantly from a normal distribution (and all parameters showed homogeneity of variance).

## Results

### Effects of the stress test

**Figure 2:**
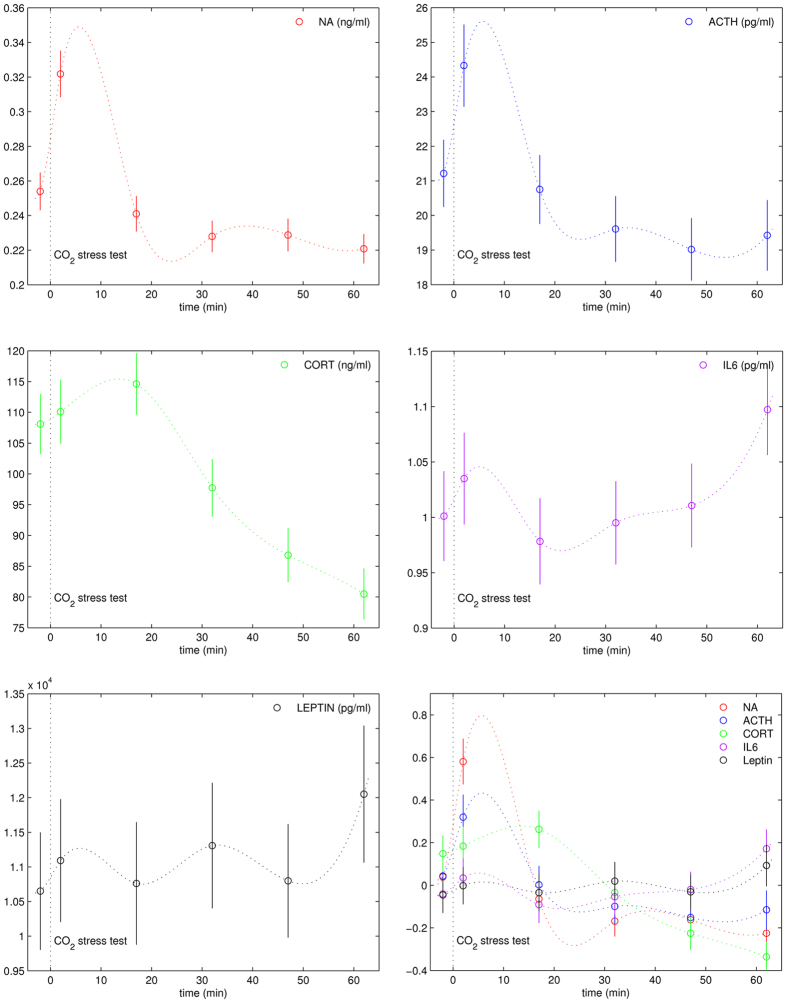
Serum levels of parameters that showed sensitivity to the CO_2_ stress test (NA, ACTH, cortisol, IL-6 and leptin), pooled across both groups, and all three times of day (*n* = 143 subjects). Mean serum levels are indicated by the circles, separately for each of the six time points (error bars represent SEM). Dotted lines are spline interpolations of mean values, thus approximating the time course of parameter levels between the measured time points. The bottom right panel shows *z*-standardized values so that responsiveness to the stress test can be compared between parameters. ACTH: adrenocorticotropic hormone; IL-6: interleukin-6; NA: noradrenaline.

The stress test elicited significant changes in serum levels of NA, ACTH, cortisol and IL-6 (Fig. 2). This was reflected in main effects of *time point* (*p* ≤ 0.001 in the MANOVAs computed for each of these serum parameters, see Methods; for details see Table 2), with effect sizes (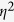) of .39 (NA), .26 (ACTH), .67 (cortisol), and .16 (IL-6). The effect of the stressor on leptin approached statistical significance (*p* < 0.05, 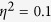 for the main effect of *time point*; see also Table 2). No effect of the stress test was observed for SIH, nor for TNF-α.

IL-6 levels were lowest at time point 3 (around 20 min after the stress test, see Fig. 2), and highest at time point 6 (i.e., at the end of the recovery period, around 1 h after the stress test; a paired *t*-test indicated that IL-6 levels were significantly higher at time point 6 than at time point 1, *p* < 0.005). Note that NA and ACTH showed maximum serum levels at time point (TP) 2, and an immediate decrease after TP 2, thus exhibiting a fast and phasic response to the stress test. Paired *t*-tests indicated significant differences between TP 1 and TP 2, as well as between TP 2 and TP 3, for both NA and ACTH (*p* < 0.0001 in each test). The spline interpolation of NA and ACTH data across time points suggests a peak latency of less than 7 min (note that this spline interpolation can only approximate the true time course of parameter levels). By contrast, cortisol levels were maximal at TP 3 (i.e., more than 15 min after the stress test), thus showing a much slower response, and a gradual decrease until the last TP (this is to be expected because both ACTH and NA are released following neuronal signalling, while cortisol release is mediated humorally). Paired *t*-tests showed that cortisol differences were not significant between TP 1 and TP 2 (*p* = 0.4), but between TP 2 and TP 3 (*p* < 0.05), and between TP 3 and TP 4 (*p* < 0.0001). At TP 6, Cortisol, NA, and ACTH levels were below levels of TP 1. Paired *t*-tests showed that cortisol, NA, and ACTH levels differed between TP 1 and TP 6 (cortisol: *p* < 0.0001, NA: *p* < 0.0005, ACTH: *p* < 0.05), probably owing to the stress and anxiety that participants felt prior to the venipuncture and the stress test.

The bottom right panel of Fig. 2 shows *z*-standardized values of parameters that exhibited significant sensitivity to the CO_2_ stress test (so that responsiveness to the stress test can be compared between parameters). These data indicate that NA had the strongest sensitivity in response to the stress test, followed by ACTH and cortisol.

### Interactions with music and mood

**Figure 3:**
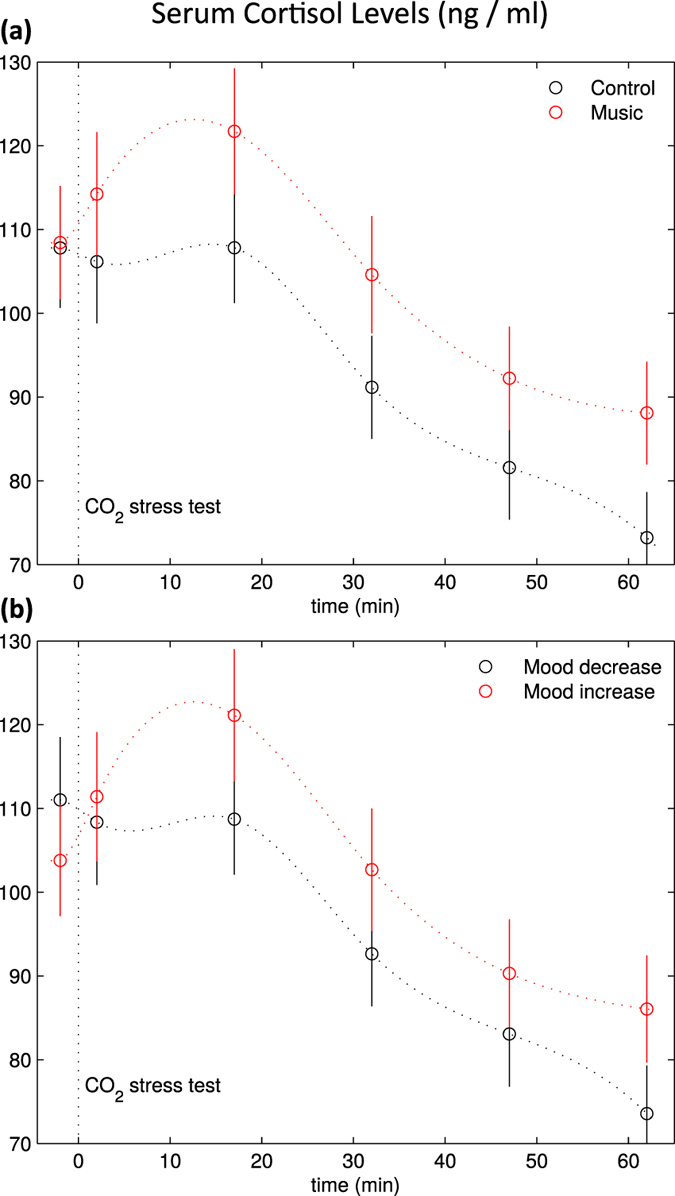
Serum levels of cortisol, separately for music group and control group (**a**), and for participants in whom mood increased vs. decreased over the course of the experiment (**b**) (as indexed by the difference between pre- and post-values of the mood questionnaire). Mean serum levels are indicated by the circles, separately for each of the six time points (error bars represent SEM). Dotted lines are spline interpolations of mean values, thus approximating the time course of parameter levels between the measured time points.

When comparing music and control groups directly with each other, the music stimulus had a significant influence on the recovery of cortisol levels, as indicated by an interaction between *time point* x *stimulus group* for cortisol (*p* < 0.02, 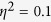; see Fig. 3a; for details see left column of Table 3). This interaction was due to cortisol levels being higher in the music group during TPs 3–6 (two-samples *t*-tests on the standardized cortisol values showed significant differences between the music and the control group for TP 3 and TP 4, *p* < 0.05, a marginally significant difference at TP 5, *p* < 0.07, and a clear difference at TP 6, *p* < 0.005). To substantiate these findings we computed a discriminant function analysis (DFA, probability for entry was *p* = 0.05, for removal *p* = 0.10) with group (music, control) as group variable, and standardized values of cortisol, NA, and ACTH as dependent variables. 61% of cross-validated cases were correctly classified (Wilks’ λ = 0.9, 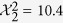, *p* < 0.005) based on two variables, which were (standardized) cortisol levels at TP 5 and TP 6.

Music was causal for a more positive mood in the music group: Participants with mood increase from pre- to post-measurements (as measured with the POMS) were clearly over-represented in the music group (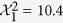, *p* = 0.001). In the music group, mood increased in 41, and decreased in 27 participants. In the control group, on the other hand, mood decreased in 47, and increased in 23 participants. Thus, not all participants in the music group experienced a positive effect of the music on their mood.

To assess effects of mood on the recovery of the stress test in more detail, participants were divided into those in whom mood increased or decreased over the course of the experiment (as indexed by the difference between pre- and post-values of the POMS). This group membership had a significant impact on the effects of the CO_2_-stressor on cortisol levels, as reflected in a significant interaction of *time point* x *POMS group* (mood increase, mood decrease) for cortisol (*F* = 4.91; *p* = 0.002; see Fig. 3b; for details see right column of Table 3). This interaction was due to group differences in cortisol at TPs 2–6, during which (standardized) cortisol levels were significantly higher in the group with mood increase compared to the group with mood decrease (two-samples *t*-tests on the standardized cortisol values showed significant differences between the music and the control group for TP 2: *p* < 0.05, TP 3 & TP 4: *p* < 0.005, TP 5: *p* < 0.01, and TP 6: *p* = 0.001).

### Valence and arousal ratings

Average valence ratings (provided on a 9-point scale ranging from −4 to +4) were *M* = −0.33 (*SD* = 1.68) in the control group (and not significantly different from zero according to a two-tailed one-sample *t*-test testing against zero, *p* > 0.1) and *M* = 2.06 (*SD* = 1.13) in the music group, the difference between groups being significant (*T*(124) = 10, *p* < 0.0001). Thus, the control stimulus was perceived as neutral, whereas the music stimulus was rated as positive. Average arousal ratings (provided on a 9-point scale ranging from 0 to 8) did not differ between control group (*M* = 2.88, *SD* = 1.29) and music group (*M* = 2.64, *SD* = 1.29).

### Circadian rhythm and sex

Data on the circadian rhythm of serum parameters and of differences between males and females are reported and discussed in the Supplementary Information. Note that neither circadian rhythm (i.e., *time of day*), nor *sex*, interacted with the factor *time-point* in any of the MANOVAs (for details see Supplementary Information)

## Discussion

The CO_2_-stressor elicited significant changes in serum levels of NA, ACTH, cortisol and IL-6, and the effect of the stressor on leptin approached statistical significance (no effect of the stress test was observed for SIH, nor for TNF-α). The effect of the stress test on NA replicates previous findings^7^, and the effect on cortisol is consonant with previous studies investigating effects of CO_2_-stress on serum cortisol levels^6^. In addition, our results provide the first evidence for effects of a CO_2_-stressor on ACTH levels. Thus, our results corroborate the notion that CO_2_-stress incites a stress response involving both the HPA axis (ACTH and cortisol) and sympathetic nervous system activity (NA). As a note of caution, it is likely that, in addition to the CO_2_-stressor, the insertion of the intravenous cannula (and anxiety associated with the venipuncture) contributed to the stress-related effects (this issue is also discussed further below).

More importantly, our results indicate that a cytokine (IL-6) was also affected by the acute stressor. This suggests that acute physiological stress affects IL-6 levels, independent of physical stress (e.g., exercise) or psychological stress (e.g., mental or social stress), extending meta-analytic data on effects of acute psychological stress on IL-6 serum levels (provided by Steptoe *et al*.)^41^. IL-6 levels were lowest at time point 3 (around 20 min after the stress test), and highest at time point 6 (i.e., at the end of the recovery period, more than 1 h after the stressor; see also Fig. 2). Again, this is consistent with the meta-analysis by Steptoe *et al*. (ref. 41), in which effects of acute psychological stress on IL-6 serum levels were stronger in studies sampling 30–120 min post-stress (compared to studies sampling immediately after a stressor). The time course of IL-6 responses has not been established previously. Our data indicate that the IL-6 peak response does not occur before 60 min after the acute physiological stressor. The findings of effects of acute stress on IL-6 levels contradict data showing that stress-related HPA and sympathetic activity simply inhibits the function of pro-inflammatory cytokines such as IL-6^10^. Thus, while immune function is depressed by chronic stress (as shown in previous studies), immune function may be enhanced by acute stress, in line with recent theories on immuno-protective effects of acute stress (see also further below)^11^. Given the well-documented immuno-suppressive function of cortisol in chronic stress, our data support the notion that a short-term peak in cortisol levels does not have immuno-inhibitory effects. The fact that we found IL-6 changes in response to a CO_2_ stressor, in contrast to a previous study that also used a CO_2_ stressor^42^, is probably due to both the larger number of subjects (143 in our study, compared to 32 in the study by van Duinen *et al*.)^42^, and the higher sensitivity of the tests we used (0.11 pg/ml in our study, compared to 1 pg/ml in the study by van Duinen *et al*.)^42^.

Another important finding is that leptin increased in response to acute stress. Even though similar results have been shown with acute mental stress (in females)^43^, our study is the first to show such an association after a physiological stressor, and in both females and males (note that leptin varied *within* subjects as a function of the stress test, that leptin levels increased although participants did not consume any food or drinks during the experimental procedure, and that BMI was balanced across study groups). Leptin is known as a mediator of obesity^44^, shows only marginal variation during daytime^45^, and is strongly modulated by psycho-immunological processes^46^. Its synthesis in adipose tissue can be induced by stress reactions, including inflammatory parameters^47^, glucocorticoids^44^, and leptin acts back on both inflammatory response^8^ and midbrain functions related to feelings of reward^48^. This multi-directional interaction has major implications for associations between obesity (i.e., eating behavior) and stress, and thus for a better psychological understanding of obesity as a major health challenge. For example, obesity increases leptin resistance^49^, and our data motivate the hypothesis that such increased leptin resistance plays a role for blunted physiological reactions to acute stress in obese individuals, perhaps even associated with dysfunctional effects of acute stress on mood and eating behavior. This issue needs to be specified in future studies.

Note that NA and ACTH showed maximum serum levels at time point (TP) 2, and an immediate decrease after TP2, thus exhibiting a fast and phasic response to the stress test. The spline interpolation of NA and ACTH data across time points suggests a peak latency of less than 7 min (note that this spline interpolation can only approximate the true time course of parameter levels). By contrast, cortisol levels were maximal at TP 3 (i.e., more than 15 min after the stress test), thus showing a much slower response, and a gradual decrease until the last TP (this is to be expected because both ACTH and NA are released following neuronal signalling, while cortisol release is mediated humorally). Knowledge about kinetics in acute stress-related processes enables in future examinations the selection of optimal time points in order to reduce costs and interference within the experimental procedure.

Cortisol, NA, and ACTH levels were lower at the end than at the beginning of the experiment. This reflects that participants were stressed already at the very beginning of the experiment, e.g. because they were anxious about the insertion of the cannula and the stress test. However, even if the acute stress is due to both stress associated with venipuncture and stress due to the CO_2_ stress, our results nevertheless clearly show effects of acute stress which are likely to be mainly due to CO_2_ stress (because CO_2_ inhalation is a considerably stronger stressor compared to a venipuncture).

The effects of the acute stress on HPA axis hormones (ACTH and cortisol), sympathetic endocrine activity (NA), inflammation (IL-6) and metabolism (leptin) emphasize interactions between stress, metabolism, and inflammation. Note that these findings were obtained following acute (short-term) stress without additional exercise or social stress. Thus, our data suggest that acute physiological stress directly incites both metabolic and immune regulation, even in the absence of exercise or social stress (as commonly used in stress experiments with humans). However, as stated above, the placement of the intravenous cannula presumably elicited psychological (anxiety) and physiological (pain) stress in several participants^38^, which contributed to the CO_2_ stress.

The *z*-standardized values of parameters that exhibited significant sensitivity to the stress test indicate that NA had the strongest sensitivity in response to the stress test, followed by ACTH and cortisol (bottom right panel of Fig. 2). This is of interest because NA synthesis in the adrenal gland medulla is not only triggered by ACTH, but also by splanchnic innervations, antagonized by parasympathetic nerves in the midbrain, and influenced by both inflammation and leptin^48^. Thus, our results reveal that NA reacts more strongly than serum cortisol and ACTH to acute stress, indicating that NA is a valid and integrative parameter for stress description, and perhaps more adequate than cortisol as a measure for a general, integrated stress response. This notion is congruent with findings from other studies showing a reliable and strong peripheral NA response to different types of stressors^50^,^51^,^52^. This is particularly important because mixed venous NA is a surrogate marker of overall sympathetic tone^53^.

When comparing music and control groups directly with each other, the music stimulus had a significant influence on cortisol levels after the stressor and during the recovery period. Cortisol levels were higher in the music group after the stressor compared to the control group. This finding is consistent with a recent study^25^, in which higher cortisol levels post-stress in a music group (compared to a silence group) were taken to reflect a facilitation of the natural physiological response to stress. Acute stress prepares the organism to deal (e.g. fight or flight) with challenges (e.g. wounding or infection) imposed by a stressor^11^. One important immunological effect of short-term stress is an induction of an increase in concentrations of circulating cytokines (such as IL-6, as also shown in our data). That is, acute stress induces an enhancement of immune function, and such immuno-protective (or immuno-enhancing) responses have been argued to be evolutionarily adaptive, e.g. because they promote efficient wound healing^11^. Notably, it has also been argued that physiological concentrations of endogenous stress hormones have immuno-enhancing effects in healthy individuals^11^. Thus, the higher cortisol levels observed in the individuals of the music group plausibly reflect the beginning of a chain of stronger immuno-enhancing effects in response to acute stress.

Importantly, the POMS data show that participants in our music group had overall more positive mood. Thus, we can rule out that the higher cortisol levels in the music group were due to higher stress levels in that group. Our suggestion that the higher cortisol in the music group is associated with immune-enhancing effects, and the lower cortisol response in the group with more negative mood associated with a suboptimal, i.e. less robust, stress response, is also consonant with findings of lower cortisol levels, and HPA hypoactivity in patients with depression (both in the basal state and in response stressors; reviewed in ref. 54). Most interestingly, our mood-induction procedure with music provides the first piece of evidence suggesting that positive mood is a causal factor for higher serum cortisol levels (and, thus, a more adequate stress response) following acute stress. The combined results show a more complicated, and sophisticated role of cortisol in the fine-tuning of the stress response than previously believed, indicating that the view that higher cortisol levels are always associated with higher stress levels must be revised.

The fact that participants with mood increase from pre- to post-measurements (as measured with the POMS) were clearly over-represented in the music group indicates that music had beneficial effects on the mood in the majority of participants in the music group. However, not all participants in the music group experienced a positive effect of the music on their mood, probably because our pre-selected music was not in accord with the musical preferences of some participants. Therefore, for future studies, we recommend to offer a selection of different music collections to the participants, so that the different sets of musical stimuli can still be controlled by the experimenters with regard to their homogeneity of emotional expression (e.g. “positive joyful music”), as well as with regard of not containing lyrics (so that it is certain that any effects are due to the music, and not due to the lyrics).

We also divided participants into those in whom mood increased or decreased over the course of the experiment (as indexed by the difference between pre- and post-values of the POMS). This group membership had a significant impact on the cortisol levels. This finding supports the association between positive mood and the cortisol response to short-term stress discussed above.

Strengths of our study include the large sample size, the measurement of several serum parameters associated with stress and immune function, the fact that two different groups underwent precisely the same experimental protocol (the only difference being the valence of the acoustical stimulus), and the use of the CO_2_ stress test, which allows a very precise examination of an acute stress reaction with regard to several different mediators.

One could argue that our results do not inform about “normal” recovery from stress without any acoustical stimulus. However, not delivering any stimulus during a recovery period bears two problems: First, it is difficult to control what participants are doing when they are not given any specific task. Secondly, when subjects are simply instructed to rest, it is likely that participants mainly engage in mind-wandering, which has been shown to have a number of uncontrollable emotional effects (both positive and negative)^55^ that potentially bias the results. Thus our control condition is likely to be even more adequate than simply letting participants relax for an hour.

A limitation of our study is reflected by the fact that cortisol, NA, and ACTH levels at TP 6 (i.e., at the end of the experiment) were below the levels at TP 1 (i.e., at the beginning of the experiment), indicating that participants were stressed already at TP 1 (e.g. due to the psychological and physiological stress associated with the insertion of the cannula and being anxious about the stress test)^38^. Thus, the data at TP 1 do not represent adequate baseline values, and the data from TP 2 and the following time points are probably not only due to the CO_2_ stressor but also the venipuncture and the anxiety of participants^38^. It might be suggested that we should have waited, e.g., 20–30 min after the insertion of the cannula, in order to measure only the effects of the CO_2_ stress test. However, our data indicate that several of the parameters show changes even one hour after the CO_2_ stress (consistent with previous findings)^38^, meaning that even a rest period of one hour would have been biased by effects due to the insertion of the cannula. Future studies might, therefore, consider adding an additional condition without CO_2_ stress test.

## Conclusions

Our results replicate effects of CO_2_ stress on NA and cortisol, and provide the first evidence for effects of this acute stressor on ACTH, IL-6, and leptin levels in humans. Thus, the combined results of our study provide evidence that acute stress incites a stress response involving HPA axis hormones, sympathetic endocrine activity, inflammation, and metabolism. Interestingly, the *z*-standardized data show that NA exhibited the strongest response to the CO_2_ stressor, suggesting that NA is perhaps more adequate than cortisol as an index for an acute general, integrated stress response. Thus, future studies on stress effects could benefit from assessing NA, rather than cortisol, serum levels as a physiological marker of stress.

Our findings were obtained following acute physiological (CO_2_) stress without additional exercise or social stress. Although the placement of the intravenous cannula presumably elicited psychological (anxiety) and physiological (pain) stress in several subjects^38^, thus contributing to the CO_2_ stress, our results suggest that acute CO_2_ stress is capable of inciting metabolic and immune regulation. Because the sex of participants did not influence the reaction to the stress test, our results give rise to the notion that sex-dependent effects of stress in other studies might have originated from the specific stressors chosen to induce stress, rather than from stress in general. The effects of the stress test on IL-6 and leptin levels suggest that both immune and metabolic functions are affected by acute physiological stress, independent of acute physical stress (e.g., exercise) or acute psychological stress (e.g., mental or social stress), and independent of chronic stress. Particularly the effect of the acute stress on leptin has important implications for the role of short-term stress in the etiology of obesity, and dysfunctional reactions to stress in obese individuals.

Finally, both stress test and recovery period were associated with higher cortisol levels in participants with positive mood (compared to participants with negative mood). Because cortisol levels are lower in patients with depression and negative mood, our data suggest that the higher cortisol response in the group with more positive mood reflects a physiologically more adequate stress response. Importantly, participants with positive mood were over-represented in the music group. This indicates that our mood-induction procedure played a causal role for the modulation of cortisol levels following the stress test, providing the first evidence that the induction of positive mood leads to more adequate stress responses, as reflected in cortisol levels.

## Additional Information

**How to cite this article**: Koelsch, S. *et al*. The impact of acute stress on hormones and cytokines, and how their recovery is affected by music-evoked positive mood. *Sci. Rep*. **6**, 23008; doi: 10.1038/srep23008 (2016).

## Supplementary Material

Supplementary Information

## Figures and Tables

**Table 1:** Descriptive statistics of the study population.

Group	*n*	Males/Females	Age (years)	Height (cm)	Weight (kg)	BMI
music	71	36/35	24.8 (2.6)	174.2 (9.9)	67.4 (12.0)	22.4 (2.4)
control	72	36/36	24.9 (2.9)	175.6 (9.5)	69.7 (10.8)	22.2 (2.6)

The sample consisted of 143 participants who were randomly assigned to either a music group or a control group. Following the CO_2_ stress, the music group listened to positive music, and the control group was presented with an auditory control stimulus. Groups were balanced with regard to group size (*n*), male/female ratio, age, body height, body weight, and body mass index (means, with standard deviation in parentheses). BMI: body mass index.

**Table 2:** Statistical values of the main effects of time point (TP), as indicated by the MANOVAs (*F*-and *p*-values, with degrees of freedom in parentheses).

	Time point (TP)
Cortisol	***F*** **=** **77.35 (3.015);** ***p *****< 0.001**
NA	***F*** **=** **35.85 (3.130);** ***p *****< 0.001**
ACTH	***F *****=** **11.62 (4.145);** ***p *****< 0.001**
IL-6	***F*** **=** **5.57 (3.151);** ***p*** **=** **0.001**
Leptin	*F* = 2.74 (4.749); *p* **=** 0.021
SIH	*F* = 0.27 (3.652); *p* **=** 0.88
TNF-α	*F* = 0.78 (4.12); *p* **=** 0.54

Main effects of *time point* reflect that serum levels differed between the six measured time points, thus indicating the effects of the CO_2_ stressor (recall that TP 1 preceded, and TP 2-6 followed the CO_2_ stress test). Significant effects (*p* < 0.005, corrected for multiple comparisons) are marked in bold. ACTH: adrenocorticotropic hormone; IL-6: interleukin-6; NA: noradrenaline; SIH: somatostatin; TNF: tumor necrosis factor.

**Table 3:** Statistical values of interactions between TP and group, as indicated by the MANOVAs reported in the main text (*F*- and *p*-values, with degrees of freedom in parentheses), pooled across all *n* = 143 participants.

	TP x Stimulus group (music, control)	TP x POMS group (increase, decrease)
Cortisol	*F* = 3.77 (3.015); *p* = 0.011	***F*** **=** **4.91 (2.986);** ***p*** **=** **0.002**
NA	*F* = 0.24 (3.130); *p* = 0.877	*F* = 0.92 (3.093); *p* = 0.436
ACTH	*F* = 1.34 (4.145); *p* = 0.252	*F* = 1.3 (4.063); *p* = 0.268
IL-6	*F* = 0.45 (3.151); *p* = 0.725	*F* = 0.79 (2.957); *p* = 0.498
Leptin	*F* = 1.62 (4.749); *p* = 0.156	*F* = 1.09 (4.838); *p* = 0.365
SIH	*F* = 1.81 (3.652); *p* = 0.132	*F* = 0.23 (3.606); *p* = 0.905
TNF-α	*F* = 1.46 (4.12); *p* = 0.211	*F* = 2.74 (4.081); *p* = 0.028

Significant interactions (*p* < 0.005, corrected for multiple comparisons) are marked in bold. ACTH: adrenocorticotropic hormone; IL-6: interleukin-6; NA: noradrenaline; SIH: somatostatin; TNF: tumor necrosis factor; TP: Time point.
