# Supplementary material for: The impact of acute stress on hormones and cytokines, and how their recovery is affected by music-evoked positive mood
Source: Sci Rep. 2016 Mar 29;6:23008. doi: 10.1038/srep23008 (PMC4810374; doi:10.1038/srep23008)
Supplement: Supplementary Information [file srep23008-s1.doc]

**SUPPLEMENTARY INFORMATION of the article:**

**The impact of acute stress on hormones and cytokines, and how their recovery is affected by music-evoked positive mood**

1. **Stefan Koelsch^1,2^, Albrecht Boehlig^3,4^, Maximilian Hohenadel^3^, Ines Nitsche^3^, Katrin Bauer^3^, & Ulrich Sack^3^**

^1^ Max Planck Institute for Human Cognitive and Brain Science, Stephanstr. 1a,
04103 Leipzig, Germany

^2^ Department of Biological and Medical Psychology, University in Bergen, Jonas Liesvei 91, 5009 Bergen, Norway

^3^ Institute of Clinical Immunology, Medical Faculty, University of Leipzig, Johannisallee 30, 04103 Leipzig, Germany

^4^ Clinic for gastroenterology and rheumatology, Medical Faculty, University of Leipzig, Liebigstr. 20, 04103 Leipzig, Germany

**Corresponding authors:**

Stefan Koelsch, Max Planck Institute for Human Cognitive and Brain Science, Stephanstr. 1a,
04103 Leipzig, Germany

phone +49 341 9940 110

e-mail koelsch@cbs.mpg.de

Ulrich Sack, Institute of Clinical Immunology, Medical Faculty, University of Leipzig, Johannisallee 30, 04103 Leipzig, Germany

phone +49 341 9725500

e-mail ulrich.sack@medizin.uni-leipzig.de

**Effects of Sex**

Leptin, cortisol, ACTH and NA levels differed significantly among males and females (main effect of *sex* with *p* ≤ .003 in each of the MANOVAs reported in the main manuscript, see also left column of Supplementary Table S1, and Supplementary Figure S1a-e): Women had higher leptin (consistent with previous investigations),1^,2^ cortisol and NA levels than men, whereas men had higher ACTH values than women. For IL-6, differences between males and females approached statistical significance (*F* = 4.69; *p* = .032), with females having higher mean IL-6 levels than men (the finding that both leptin and IL-6 levels appear to be higher in females than males is consistent with findings showing that levels of leptin and pro-inflammatory cytokines such as IL-6 are usually correlated).3 *Sex* did not interact with the factor *time-point* in any of the MANOVAs (see right column of Supplementary Table S1).

The observed differences between males and females are known to have functional consequences: females exhibit higher circulating leptin and greater cardiovascular responses to stress than males.2 Interestingly, in our setting the sex of participants did not influence the reaction to the stress test (no significant interactions between *time point* and *sex* for any of the serum parameters; see Table S1). This suggests that sex-dependent effects of stress in other studies might have depended less on stress in general, but on the specific stressors chosen to induce stress. In our study, we used a physiological stressor (stress was induced by CO_2_), which acts directly and independently of confounding factors such as psychological or physical stress.

|  | **Sex** | **TP x Sex** |
| --- | --- | --- |
| **Cortisol** | ***F*=31.31 (1); *p* < .001** | *F*=1.99 (3.015); *p* = .115 |
| **NA** | ***F*=9.45 (1); *p* = .003** | *F*=1.28 (3.130); *p* = .281 |
| **ACTH** | ***F*=25.26 (1); *p* < .001** | *F*=1.08 (4.145) ; *p* = .369 |
| **IL-6** | *F*=4.69 (1); *p* = .032 | *F*=1.01 (3.151) ; *p* = .391 |
| **Leptin** | ***F*=97.06 (1); *p* < .001** | *F*=1.44 (4.749) ; *p* = .211 |
| **SIH** | *F*=1.75 (1); *p* = .189 | *F*=2.8 (3.652); *p* = .03 |
| **TNF-α** | *F*=.03 (1); *p* = .854 | *F*=.72 (4,12); *p* = .581 |

**Supplementary Table S1: Main effect of *sex* and interactions between time point and sex (within the MANOVAs reported in the main manuscript).** The table provides *F*- and *p*-values, with degrees of freedom in parentheses. Main effects of *sex* indicate differences in serum levels between males and females. Significant effects (*p* < .005, corrected for multiple comparisons) are marked in bold. ACTH: adrenocorticotropic hormone; IL-6: interleukin-6; NA: noradrenaline; SIH: somatostatin; TNF: tumor necrosis factor; TP: Time point.

**Effects of Circadian rhythm**

Independently of the CO_2_ stress test, both cortisol and SIH showed an effect of time of day (main effect of *time of day* with *p* < .001 in each of the MANOVAs reported in the main manuscript, see also left column of Supplementary Table S2 and Supplementary Figure S1f-i). Cortisol was significantly lower at 5:00 p.m. than at the other two times of day, consistent with findings showing that, during daytime, cortisol levels are normally lowest between and 5 and 6 p.m.4 SIH values were higher at 2:00 p.m. compared to 11:00 a.m. and 5:00 p.m. For ACTH and IL-6, differences in time of day approached statistical significance (p < .05). ACTH levels tended to be higher at 2:00 p.m. compared to 5:00 p.m. IL-6 levels tended to be lower at 11:00 a.m. than at the other two times of day. *Time of day* did not interact with the factor *time-point* in any of the MANOVAs (see right column of Supplementary Table S2).

To our knowledge, these are the first results showing a circadian rhythm of somatostatin in humans, consonant with animal data.5 NA levels did not vary between the three times of day in our study, probably due to the fact that NA shows the strongest diurnal decrease during night time.6

Circadian rhythms have been shown in humans for ACTH, cortisol and noradrenalin,7 interleukin-6,8 leptin,9 and various other hormones.10 However, to our knowledge there is lack of information about a possible circadian rhythm in the production of somatostatin in humans (for animal studies see REF. 5), and no information is available with regard to TNF-α serum levels (for TNF-α secretion rhythms in monocytes see REF. 11.

|  | **Time of day** | **TP x Time of Day  (11 a.m., 2 p.m., 5 p.m.)** |
| --- | --- | --- |
| **Cortisol** | ***F*=8.96 (2); *p* < .001** | *F*=2.52 (6.029); *p* = .021 |
| **NA** | *F*=1.88 (2); *p* = .158 | *F*=.21 (6.26); *p* = .978 |
| **ACTH** | *F*=3.95 (2); *p* = .022 | *F*=2.41 (8.291); *p* = .014 |
| **IL-6** | *F*=3,59 (2); *p* = .031 | *F*=.39 (6.302) ; *p* = .895 |
| **Leptin** | *F*=.63 (2) ; *p* = .532 | *F*=1.2 (9.497) ; *p* = .29 |
| **SIH** | ***F*=10.39 (2); *p* < .001** | *F*=.86 (7.304); *p* = .539 |
| **TNF-α** | *F*=2.39 (2); *p* = .098 | *F*=.77 (8.24); *p* = .634 |

**Supplementary Table S2: main effect of *time of day* and interactions between *time point* and *time of day* (within the MANOVAs reported in the main manuscript).** The table provides *F*- and *p*-values, with degrees of freedom in parentheses. Main effects of *time of day* indicate differences in serum levels between the three measured times of day (11 a.m., 2 p.m., and 5 p.m.), and thus circadian variations during daytime. Significant effects (*p* < .005, corrected for multiple comparisons) are marked in bold. ACTH: adrenocorticotropic hormone; IL-6: interleukin-6; NA: noradrenaline; SIH: somatostatin; TNF: tumor necrosis factor; TP: Time point.


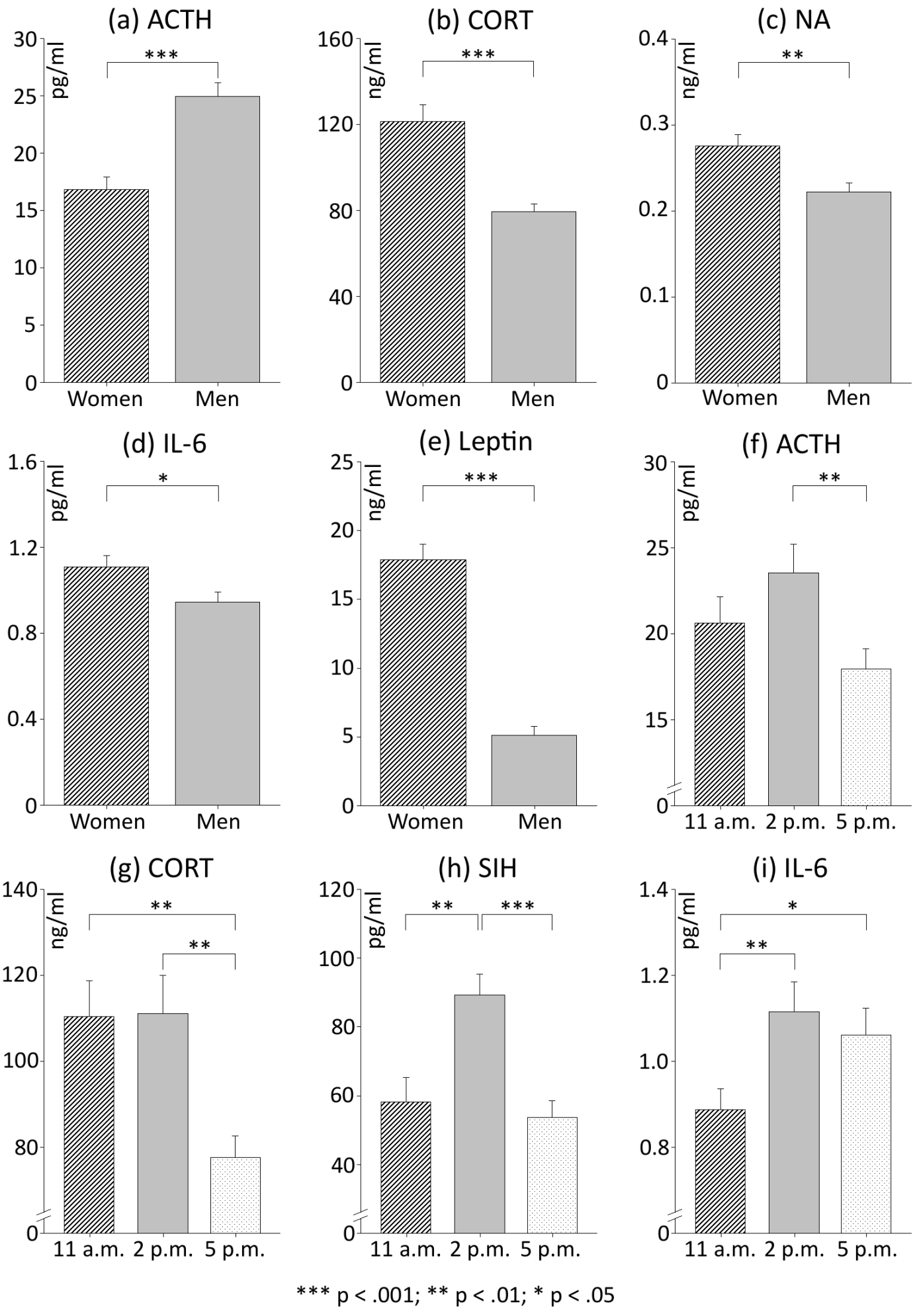


**Supplementary Figure S1**. Main effects of sex (a-e) and time of day (f-i). Error bars represent SEM. Data of panels a-e are pooled across both groups and across all three times of day in (72 males, 71 females). Data of panels f-i are pooled across both groups and across males and females (11 a.m.: 48 subjects; 2 p.m.: 47 subjects; 5 p.m.: 48 subjects).

**REFERENCES**

1. Saad, M. F. *et al.* Sexual dimorphism in plasma leptin concentration 1. *The Journal of Clinical Endocrinology & Metabolism* **82**, 579–584 (1997).

2. Brydon, L. *et al.* Circulating leptin and stress-induced cardiovascular activity in humans. *Obesity* **16**, 2642–2647 (2008).

3. Saucillo, D. C., Gerriets, V. A., Sheng, J., Rathmell, J. C. & MacIver, N. J. Leptin metabolically licenses t cells for activation to link nutrition and immunity. *The Journal of Immunology* **192**, 136–144 (2014).

4. Chan, S. & Debono, M. Review: Replication of cortisol circadian rhythm: new advances in hydrocortisone replacement therapy. *Therapeutic advances in endocrinology and metabolism* **1**, 129–138 (2010).

5. Berelowitz, M., Perlow, M. J., Hoffman, H. J. & Frohman, L. A. The diurnal variation of immunoreactive thyrotropin-releasing hormone and somatostatin in the cerebrospinal fluid of the rhesus monkey. *Endocrinology* **109**, 2102–2109 (1981).

6. Linsell, C., Lightman, S., Mullen, P., Brown, M. & Causon, R. Circadian rhythms of epinephrine and norepinephrine in man. *The Journal of Clinical Endocrinology & Metabolism* **60**, 1210–1215 (1985).

7. Cutolo, M., Buttgereit, F. & Straub, R. Regulation of glucocorticoids by the central nervous system. *Clinical and experimental rheumatology* **29**, S–19 (2010).

8. Vgontzas, A. N. *et al.* Il-6 and its circadian secretion in humans. *Neuroimmunomodulation* **12**, 131–140 (2005).

9. Patton, D. F. & Mistlberger, R. E. Circadian adaptations to meal timing: neuroendocrine mechanisms. *Frontiers in neuroscience* **7** (2013).

10. Kalsbeek, A. & Fliers, E. Daily regulation of hormone profiles. *Circadian clocks*, 185–226. Springer (2013).

11. Keller, M. *et al.* A circadian clock in macrophages controls inflammatory immune responses. *Proceedings of the National Academy of Sciences* **106**, 21407–21412 (2009).
